# Efficacy and Safety of Fusion Imaging in Radiofrequency Ablation of Hepatocellular Carcinoma Compared to Ultrasound: A Meta-Analysis

**DOI:** 10.3389/fsurg.2021.728098

**Published:** 2021-12-06

**Authors:** Tao Jie, Feng Guoying, Tang Gang, Shi Zhengrong, Li Maoping

**Affiliations:** ^1^Department of General Surgery, Chongqing Medical University, Chongqing, China; ^2^Department of General Surgery, The First Affiliated Hospital of Chongqing Medical University, Chongqing, China; ^3^Department of Ultrasound, The First Affiliated Hospital of Chongqing Medical University, Chongqing, China

**Keywords:** curative effect, fusion imaging, meta-analysis, security, ultrasound

## Abstract

**Background:** Radiofrequency ablation (RFA), generally performed under real-time guidance of ultrasound which is safe and effective, is a common minimally invasive therapy for treating hepatocellular carcinoma. Fusion imaging (FI) is a newly developed imaging method, which integrates CT/MRI accurate imaging and matches the characteristics of real-time ultrasound imaging, thereby providing a new approach to guide tumor ablation therapy. However, the efficacy and safety of FI as opposed to ultrasound in tumor ablation remains unclear.

**Objective:** The present study sought to evaluate the difference in the efficacy and safety between FI and ultrasound in radiofrequency surgery for the treatment of hepatocellular carcinoma through a metaanalysis.

**Materials and Methods:** Searching for studies comparing the efficacy and safety of FI and ultrasound in radiofrequency of hepatocellular carcinoma in PubMed, Embase, and Cochrane Library databases for articles published until April 2021. Random or fixed effect models were used for statistical analysis. Metaanalysis and sensitivity analysis were used on the included studies.

**Results:** A total of six studies met predefined inclusion criteria, and were finally included in the analysis. Sensitivity and subgroup analyses, based on predetermined patient characteristics, allowed minimization of bias. In the RFA of hepatocellular carcinoma, FI decreased 1-year overall survival (OS) when compared with ultrasound. But FI was not significantly different from ultrasound in terms of technical efficiency, 1-, 2-, and 3-year local tumor progression (LTP), complications, as well as 2-year OS. Subgroup analysis, based on tumor mean diameter, showed that FI reduced the rate of 1- and 2-year LTP in patients with tumors of mean diameter ≥15 mm when compared with ultrasound. Moreover, operative complications could be reduced in patients with tumor mean diameter <15 mm using FI, compared with ultrasound.

**Conclusion:** Overall, these results showed that FI may have some effects on improving efficacy and safety of thermal ablation in HCC patients, relative to ultrasound. However, it may be a more effective method for managing large lesions, as well as those that are difficult to ablate. Further large-scale and well-designed randomized controlled trials are needed to validate these findings.

## Introduction

Radiofrequency ablation (RFA) is a safe and effective method for treating patients with early hepatocellular carcinoma who cannot tolerate surgery or are reluctant to undergo surgery (1, 2), while imaging holds the key to the curative effect and prognosis of frequency ablation (3). On the other hand, ultrasound (US) remains the most commonly used imaging technique (4, 5) due to its economic convenience, nonionizing radiation, and real-time characteristics (6, 7). However, the imaging of ultrasound is relatively fuzzy in the face of lesions <2 cm (8), isoechoic, located in the center or top of the liver, as well as interference from adjacent structures and tissues (9). Therefore, contrast-enhanced ultrasound (CEUS) is required. However, the positioning ability for CEUS is also limited for tumors with poor blood supply and the situation where it is difficult to evaluate the ablation range, which may lead to tumor residue (10). In addition, tumors that are not visible in ultrasound, remain a major challenge during RFA (8). Therefore, RFA under ultrasound alone is constrained by numerous limitations.

Advancements in computer graphics, 3D image processing technology, and the emergence of fusion imaging (FI) have all improved RFA (11). FI, which overlaps images from different image sources and combines real-time images of ultrasound with the high resolution of CT/MRI (9), has been developed. Notably, this technique is more accurate than ultrasound alone in identifying target lesions, thereby allowing ablation of that are invisible or ablate tumors that are difficult to ablate (8). In addition, FI can also determine the ablation edge and evaluate treatment response in real time (12), has excellent efficacy and safety (13, 14), and may become an important imaging technique for RFA of hepatocellular carcinoma. In fact, the technique can also be used in needle biopsy for disease diagnosis (15), and is also a promising application in prostate (16), liver (17), heart (18), and brain diseases (19), among others.

However, published studies have yielded conflicting results with regards to efficacy and safety of FI relative to that of ultrasound. While some studies have shown that FI is more superior than ultrasound (20, 21), others have found no significant differences in the two technologies (22, 23). Therefore, the present metaanalysis was designed to systematically evaluate efficacy and safety of FI relative to ultrasound in radiofrequency surgery for the treatment of hepatocellular carcinoma. Specifically, patients with hepatocellular carcinoma undergoing RFA were selected as the research objects, and technical efficiency, local tumor progression (LTP), and complications [thoracic hemorrhage, biliary injury, so on (24)] were taken as the main evaluation indexes, whereas survival (OS) was considered the secondary evaluation.

## Materials and Methods

### Literature Search

**Figure 1:**
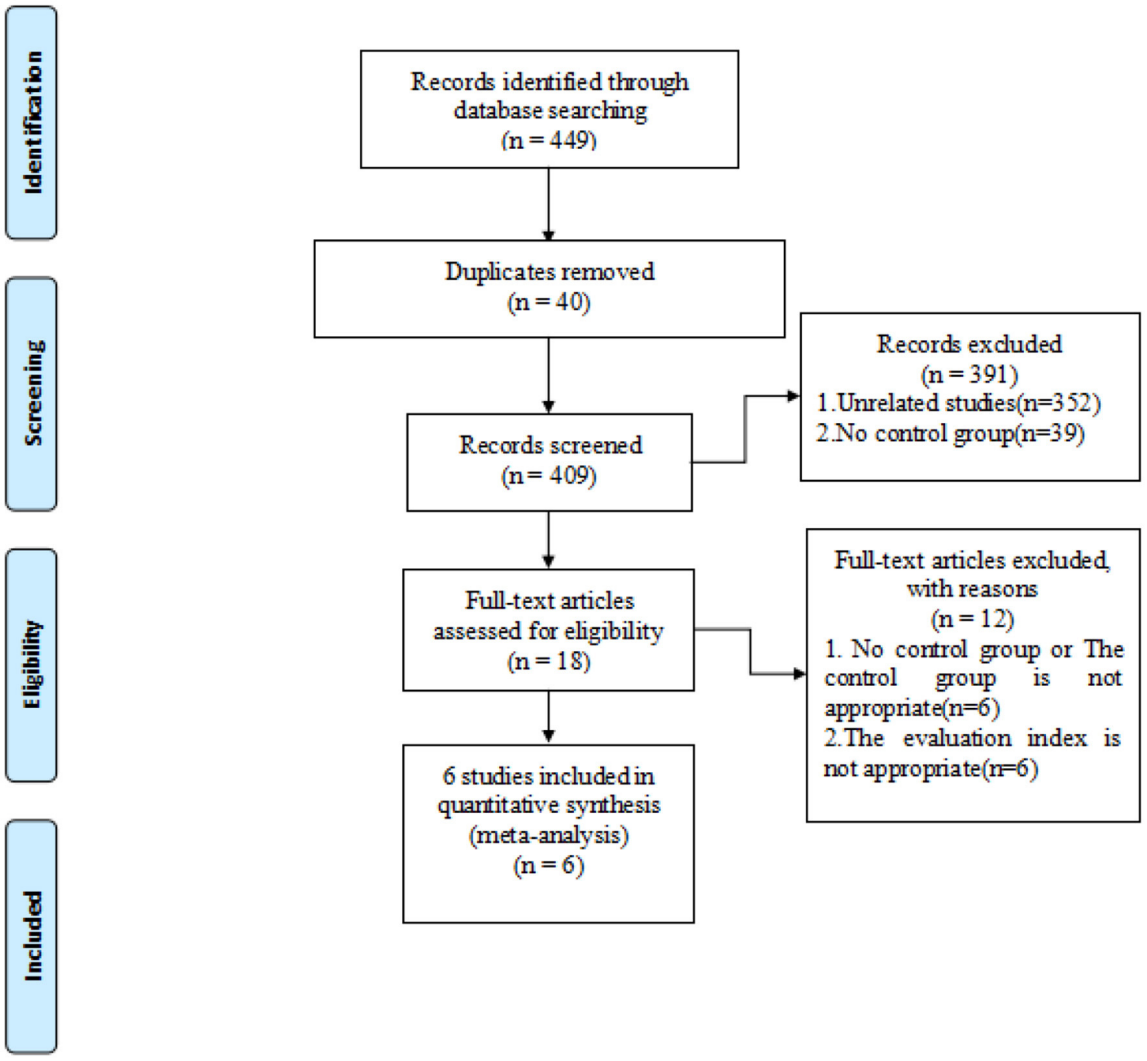
PRISMA flow diagram of study selection process.

Articles from PubMed, Embase, and Cochrane library databases were searched and relevant articles were retrieved. Search strategy involved the following keywords: hepatocellular carcinoma, RFA, FI, and ultrasound. Due to the relatively new development of FI in hepatocellular carcinoma radiofrequency, studies published were screened until April 1, 2021, and studies published after that date were not included. References of selected literatures were also screened to prevent the omission of relevant studies. In the initial screening, we read the title and abstract to determine if it met our inclusion criteria. The available full-text articles were then reviewed as described (Figure 1). Specifically, two reviewers (Tao and Tang) conducted literature retrieval and data extraction, and any questions were resolved through discussion with other reviewers (Feng and Shi and Li).

### Inclusion and Exclusion Criteria

Inclusion criteria were: (1) Studies to compare application of FI and ultrasound or FI and CEUS in RFA of patients with nonrecurrent hepatocellular carcinoma; (2) reported results included at least one of the technical efficiencies, LTP, complications, and OS. Exclusion criteria were: (1) non-English papers or repetitive articles; (2) Unpublished data or gray literature including conference abstracts, dissertation, brief reports, book chapters, editorials, and patents. Any discrepancies regarding selection of a qualified article were resolved through discussion or consultation with other reviewers (Feng and shi and li).

### Data Extraction

To reduce chances of human error, data extraction for each study was performed independently by two reviewers (Tao and Tang) using a developed form. Data collected included, name of the first author, study design, country, and year of publication, sample diameter, participants' gender, and their mean age, liver function (Child-Pugh class), tumor diameter, type of intervention in the control group, and main outcomes. There were minimal disagreements between the two researchers with regards to data extraction or quality assessment, and these issues were resolved through discussion and consensus.

### Quality Assessment

Since the included articles included both a cohort study and a randomized controlled studies, the two reviewers (Tao and Tang) evaluated the quality of each cohort study using the Newcastle–Ottawa quality assessment scale, whereas that of randomized controlled studies was performed using the risk of bias assessment scale in the Cochrane manual of systematic evaluation of interventions. If both reviewers had different views on the results, following the separate assessment, other researchers (Feng and Shi and Li) were called upon to help to reach a consensus. Quality of the cohort studies was assessed based on three factors, namely selection, comparability and outcome. On the other hand, risk of bias in randomized controlled studies was assessed based on seven criteria, namely randomization, distribution hiding, blindness of participants and operators, detection blindness, incomplete data, selective reporting, and other biases.

### Statistical Analyses

The primary endpoints in the meta-analysis included technical efficiency, 1-, 2-, and 3-year LTP, and complications, whereas secondary endpoints were 1- and 2-year OS. Heterogeneity among studies was assessed using the *I*-square (*I*^2^) and *Q* tests. When *I*^2^ < 50% and *P* > 0.1 in the *Q*-value test, a fixed-effect model was applied. Otherwise, the random effects model was employed. Assessment of potential sources of interstudy heterogeneity was performed using subgroup analyses, based on baseline tumor mean diameter (tumor mean diameters ≥15 and <15 mm, the data was obtained by looking at all the literatures) and control type (US and CEUS). The proportion of each study to the overall outcome was assessed using sensitivity analysis, while publication bias was evaluated by grade correlation based on Begg and regression asymmetry test of Egger. Statistical analyses were performed using Stata 15.1 software (Statacorp, College Station, Texas, USA) and Review Manager5.3, by two investigators (Tao and Tang), and reviewed by the other researchers (Feng and Shi and Li).

## Results

### Literature Search

The selection process of the present study is shown using the PRISMA flow diagram (Figure 1). Search strategy resulted in a total of 449 records, of which 40 were duplicates and were subsequently eliminated. The remaining 409 records were screened by title/abstract, and 18 selected for full-text assessment. An additional 12 records did not meet our inclusion criteria, leaving a final six full-text articles for metaanalysis (10, 21–23, 25, 26).

### Characteristics of the Included Studies

Characteristics of the included studies, comprising six articles with 1,158 patients aged between 29 and 88 years from Asia, are summarized in Table 1. The studies were three arm tests (22, 26), treating them as four two-arm tests [Huang (22) (1): contrasting FI combined with CEUS and CEUS; Huang (22) (2): contrasting FI and CEUS; (26) (1): contrasting FI combined with CEUS and CEUS; (26) (2): contrasting FI and CEUS]. Six studies reported 1- and 2-year LTP, four reported on 3-year LTP, while eight analyzed complications of RFA. Moreover, six and two studies were cohort and randomized controlled studies, respectively. Five cohort studies were assessed using the Newcastle–Ottawa quality assessment scale (Table 2), whereas one randomized controlled study was assessed using the Cochrane risk bias assessment tool (Table 3).

**Table 1:** Characteristics of included trials.

**References**	**Country**	**Sample diameter (Number of cases, number of tumor)**		**Study type**	**Age**	**Gender (M/F)**	**Intervention**		**Child-Pugh class (A/B)**	**Tumor mean diameter (mm)**	**Outcomes**
		**case**	**Control**				**case**	**Control**			
Ma et al. (21)	China	97, 110	83, 90	Cohort study	52.3	160/20	CT/MRI-US	US	162/18	18.9 (10–48)	Technical efficiency, LTP, RFS, OS, complications
You et al. (23)	China	14, unknown	13, unknown	Cohort study	54	25/2	MRI-US	US	26/1	20.1 (9–49)	LTP, complications
Toshikuni et al. (25)	Japan	25, unknown	20, unknown	Cohort study	73.4	22/23	CT/MRI-US	US	38/7	19 (<25)	complications
Minami et al. (26)	Japan	37, 57	192, 344	Cohort study	69	161/68	CT/MRI-CEUS	CEUS	Unknown	14.7 (5–60)	Technical efficiency, LTP, RFS, OS, complications
Minami et al. (26)	Japan	123, 155	192, 344	Cohort study	70.1	229/86	CT/MRI-US	CEUS	Unknown	14.7 (5–60)	Technical efficiency, LTP, RFS, OS, complications
Ju et al. (10)	China	98, 126	92, 120	Cohort study	53.7	181/9	CT/MRI-CEUS	CEUS	166/24	20.5 (10–60)	Technical efficiency, LTP, RFS, OS, complications
Huang et al. (22)	China	124, 153	125, 150	Randomized controlled trial	53.6	226/23	CT/MRI-CEUS	CEUS	238/11	18.9 (10–49)	Technical, efficiency, LTP, RFS, OS, complications
Huang et al. (22)	China	125, 153	125, 150	Randomized controlled trial	54.6	227/23	3DUS-CEUS	CEUS	239/11	18.7 (10–44)	Technical efficiency, LTP, RFS, OS, complications

*LTP, local tumor progression rate; RFS, recurrence-free survival; OS, overall survival*.

**Table 2:** Outcome of assessment of the quality of nonrandomised studies using the Newcastle-Ottawa scale study.

**Study**	**Selection**			**Comparability**			**Outcome**			**Total score**
	**Representativeness of the exposed cohort**	**Selection of non-exposed cohort**	**Ascertainment of exposure**	**Outcome not presented at the start**	**Age and sex**	**Additional factors**	**Assessment of outcome**	**Follow-up long enough**	**Adequacy of follow up**	
Minami et al. (26)	–	*	*	*	*	*	*	*	*	8/9
Toshikuni et al. (25)	*	*	*	*	*	*	–	*	*	8/9
Ju et al. (10)	*	*	*	*	*	*	*	*	*	9/9
Ma et al. (21)	*	*	*	*	*	–	*	*	*	8/9
You et al. (23)	*	*	*	*	*	–	*	*	*	8/9

A single asterisk (*) indicates 1 score, and dash (–) indicates 0 score.

**Table 3:** Risk of bias table.

**Bias**	**Authors' judgement**	**Support for judgement**
Random sequence generation (selection bias)	Low risk	Random number
Allocation concealment (selection bias)	High risk	Doctors and data collectors know the results of the assignment
Blinding of participants and personnel (performance bias)	High risk	No blinded
Blinding of outcome assessment (detection bias)	Unclear risk	Insufficient information to judge
Incomplete outcome data (attrition bias)	Low risk	Data is balanced between groups
Selective reporting (reporting bias)	Low risk	Non-selective reporting
Other bias	Low risk	There was no obvious other bias

### Effect on Technical Efficiency

**Figure 2:**
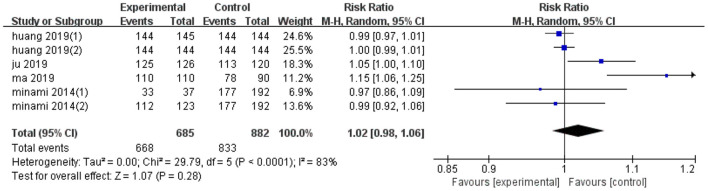
Forest plot of the effects of ultrasonic image fusion on technical efficiency.

Pooled effect diameter analysis did not reveal any significant differences in the technical efficiency between ultrasonic image fusion and the control group, across the six included trials (RR, RE: 1.02; 95% CI: 0.98, 1.06, *p* = 0.28; Figure 2). However, there was significant heterogeneity between the effect diameter of included studies (*I*^2^ = 83%, *p* < 0.0001). Moreover, subgroup analysis, based on mean diameter (<15 mm) revealed no heterogeneity (*I*^2^ = 0.0%, *p* = 0.77). Results of sensitivity analysis, used to examine the effect of each study on pooled effect diameter, revealed that exclusion of Ma's study (21) from the analysis altered the overall effect diameter (RR, RE:1; 95% CI: 0.99, 1.02, *p* = 0.84).

Moreover, no evidence of significant publication bias was found across the included studies with regards to technical efficiency (*p* = 0.81, Begg's test and *p* = 0.65, Egger's test). Results of subgroup analyses on technical efficiency are presented in Table 4.

**Table 4:** Subgroup analysis to assess the effects of ultrasonic image fusion on radiofrequency ablation.

**Indicators**	**Subgrouped by**	**The number of studies**	**Effect diameter**	**95%CI**	***I*^2^ (%)**	***P* for between subgroup heterogeneity**
Technical efficiency	Baseline mean diameter				39.3	0.2
	≥15 mm	4	1.04	0.98, 1.1	95	<0.0001
	<15 mm	2	0.98	0.93, 1.04	0	0.77
1-year LTP	Baseline mean diameter				77.8	0.03
	≥15 mm	4	0.48	0.24, 0.95	42	0.16
	<15 mm	2	1.34	0.69, 2.62	0	0.67
2-year LTP	Baseline mean diameter				84.3	0.01
	≥15 mm	4	0.45	0.27, 0.76	40	0.17
	<15 mm	2	1.34	0.69, 2.62	0	0.67
Complications	Baseline mean diameter				77.7	0.03
	≥15 mm	6	0	−0.02, 0.02	33	0.19
	<15 mm	2	−0.07	−0.12, −0.01	0	0.37
	Control group				0	0.74
	US	3	−0.04	−0.15, 0.07	76	0.01
	CEUS	5	−0.02	−0.05, 0.01	68	0.01

### Effect on LTP

**Figure 3:**
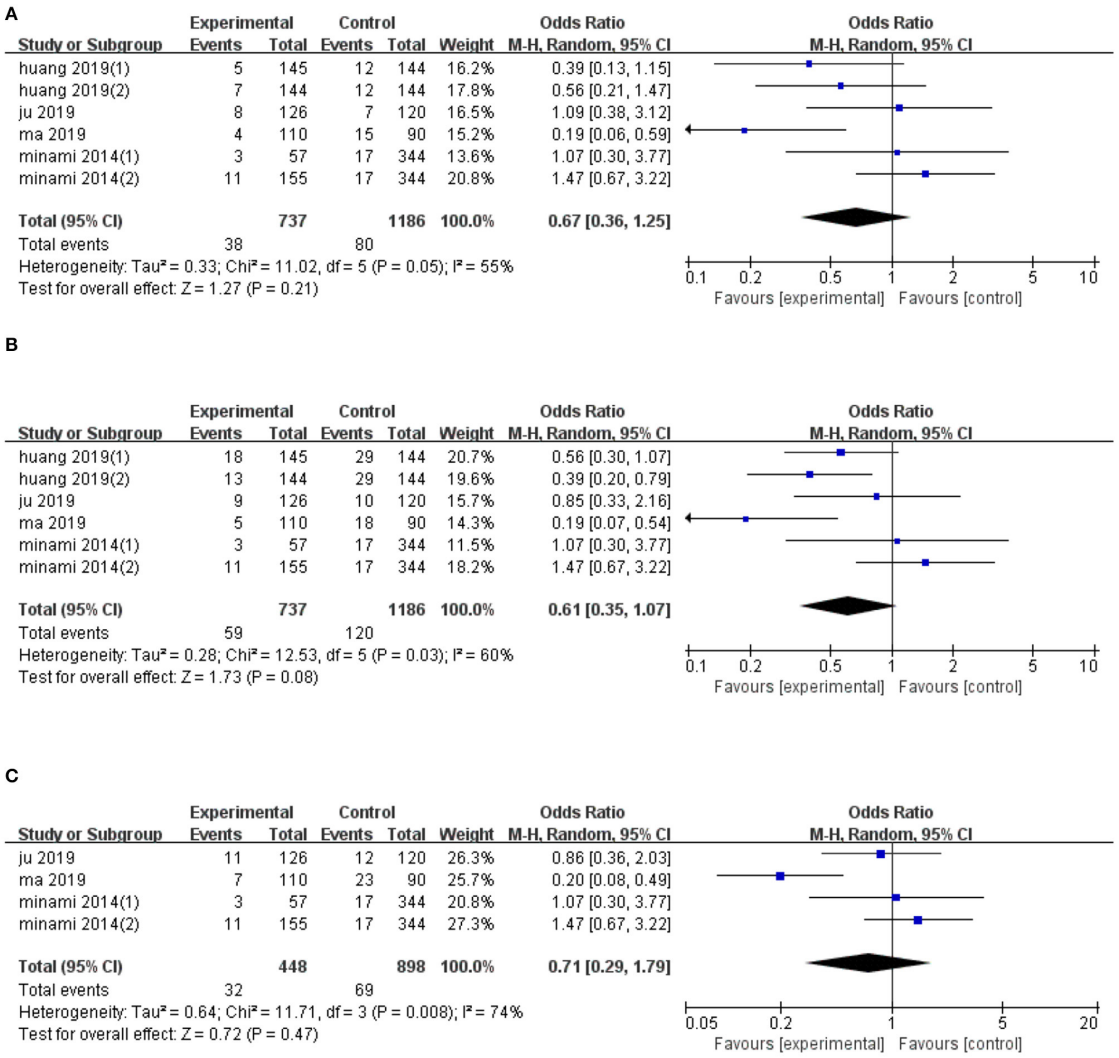
Forest plot of the effects of ultrasonic image fusion on local tumor progression. **(A)** 1-year LTP, **(B)** 2-year LTP, and **(C)** 3-year LTP.

Six trials reported data on 1-year LTP, and their pooled effect diameter based on ultrasonic image fusion, relative to the control group was (OR, RE: 0.67; 95% CI: 0.36, 1.25, *p* = 0.21), with a heterogeneity (*I*^2^ = 55%, *p* = 0.05; Figure 3A). When the metaanalysis was subgrouped by mean diameter, heterogeneity was attenuated in studies with ≥15 mm (*I*^2^ = 42%, *p* = 0.16, test for overall effect: *z* = 2.1, *p* = 0.04) and in studies with <15 mm (*I*^2^ = 0%, *p* = 0.67). Notably, we found significant differences between subgroup heterogeneity (*I*^2^ = 77.8%, *p* = 0.03). Results from sensitivity analysis revealed that exclusion of Ma's study (21) altered the overall effect diameter (OR, RE: 0.86; 95% CI: 0.52, 1.41, *p* = 0.55).

The pooled mean difference for the six datasets, with regards to the effect of ultrasonic image fusion on 2-year LTP, was (OR, RE: 0.61; 95% CI: 0.35, 1.07, *p* = 0.08) relative to ultrasonoscopy (Figure 3B), with a heterogeneity of (*I*^2^ = 60.0%, *p* = 0.03). When the metaanalysis was subgrouped by mean diameter, heterogeneity was attenuated in studies with ≥15 mm (*I*^2^ = 40%, *p* = 0.17, test for overall effect: *z* = 2.99, *p* = 0.003), and in studies with <15 mm (*I*^2^ = 0%, *p* = 0.67). Then, there was a significant between-subgroup heterogeneity (*I*^2^ = 84.3%, *p* = 0.01). To examine the effect of each study on pooled effect diameter, we performed sensitivity analyses and found that (26) (2)'s study (26) altered the overall effect diameter (OR, RE: 0.50; 95% CI: 0.31, 0.82, *p* = 0.007).

We also examined the effect of ultrasonic image fusion on 3-year LTP in four clinical trials. Overall, metaanalysis revealed no significant effects of the ultrasonic image fusion on 3-year LTP, relative to the control group (OR, RE: 0.71; 95% CI: 0.29, 1.79, *p* = 0.47), with heterogeneity across studies (*I*^2^ = 74%, *p* = 0.008; Figure 3C). Moreover, sensitivity analysis showed that excluding Ma's study (21) from the analysis changed the overall effect (OR, RE: 1.14; 95% CI: 0.67, 1.93, *p* = 0.63).

Begg's and Egger's tests did not reveal evidence of publication bias for LTP across 1-year (*p* = 0.26 and *p* = 0.272, respectively), 2-year (*p* = 1.00 and *p* = 0.915, respectively), and 3-year (*p* = 0.73 and *p* = 0.901, respectively) periods. The result of subgroup analysis on LTP are presented in Table 4.

### Effect on Complications

**Figure 4:**
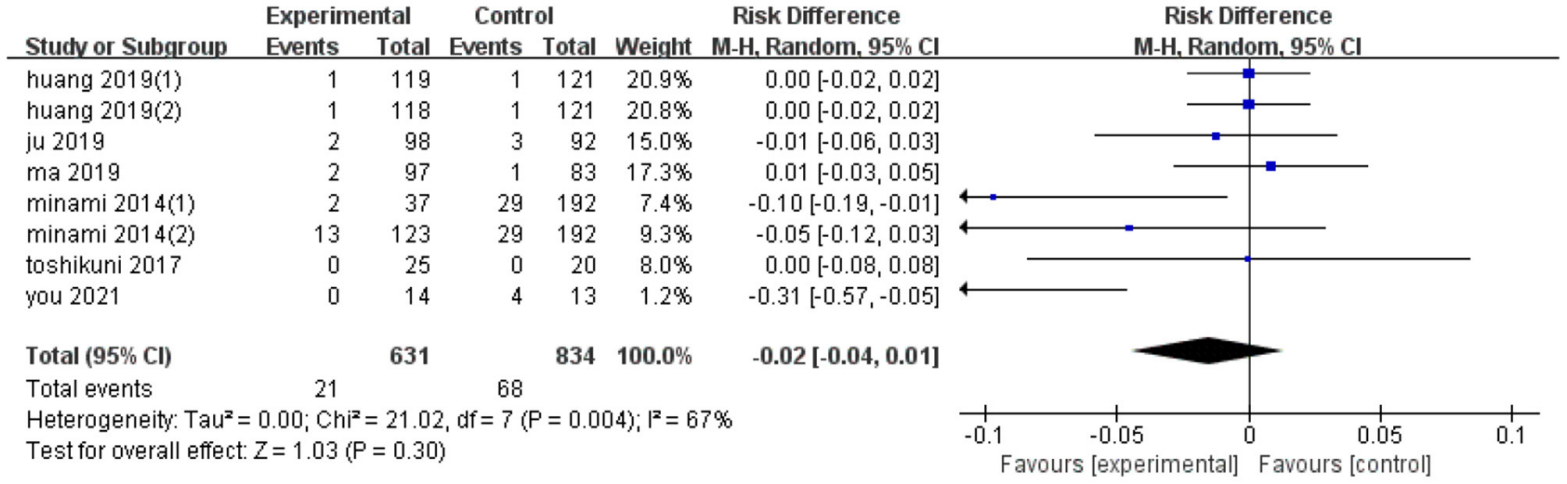
Forest plot of the effects of ultrasonic image fusion on complications.

Eight trials reported the effect of ultrasonic image fusion on complications. Metaanalysis showed that ultrasonic image fusion had no significant decrease on the complications (RD, RE: −0.02; 95% CI: −0.04, 0.01, *p* = 0.3; Figure 4), with a heterogeneity of (*I*^2^ = 67%, *p* = 0.004). When the metaanalysis was subgrouped by mean diameter, heterogeneity was attenuated in studies with ≥15 mm (*I*^2^ = 33%, *p* = 0.19) and studies with <15 mm (*I*^2^ = 0%, *p* = 0.37, test for overall effect: *z* = 2.29, *p* = 0.02). Then, there was a significant between-subgroup heterogeneity (*I*^2^ = 77.7%, *p* = 0.03). In addition, sensitivity analysis revealed that the study by You et al. (23) had a significant influence on the effect value (RD, RE: −0.01; 95% CI: −0.03, 0.01, *p* = 0.45).

Notably, there was no publication bias, possibly due to the small sample diameter and short follow-up times reported in the studies. Results of subgroup analyses on complications are presented in Table 4.

### Effect on Overall Survival

**Figure 5:**
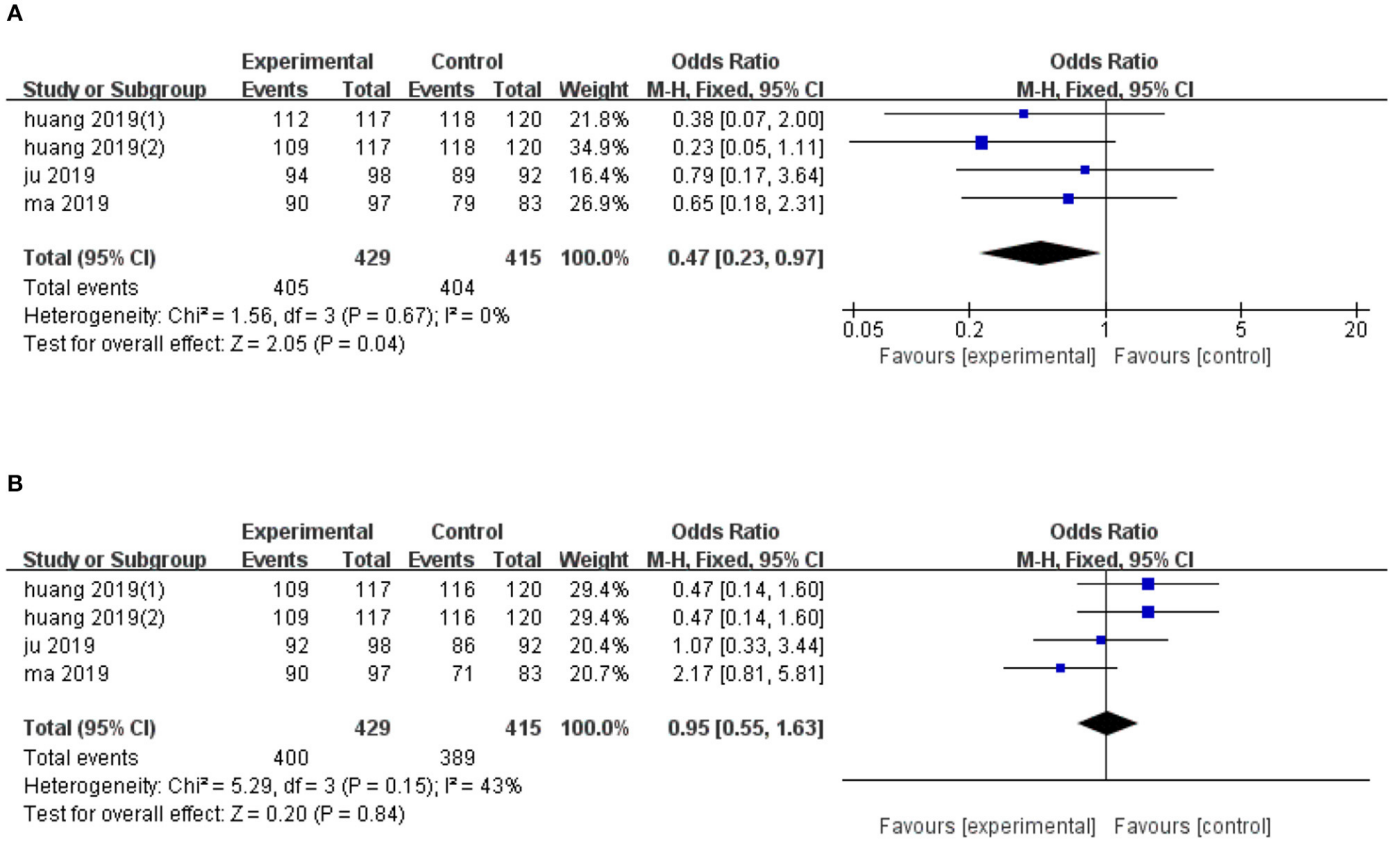
Forest plot of the effects of ultrasonic image fusion on overall survival. **(A)** 1-year OS and **(B)** 2-year OS.

Quantitative analysis of overall survival, across four trials, revealed significantly lower 1-year overall survival in the ultrasonic image fusion, relative to the control group (OR, FE: 0.47; 95% CI: 0.23, 0.97, *p* = 0.04), with no evidence of heterogeneity across the studies (*I*^2^ = 0%, *p* = 0.67; Figure 5A). Moreover, ultrasonic image fusion had no effect on 2-year overall survival of patients across four studies that evaluated this technique, relative to controls (OR, FE: 0.95; 95% CI: 0.55, 1.63, *p* = 0.85; Figure 5B). Low heterogeneity across studies was seen (*I*^2^ = 43%, *p* = 0.15).

Begg's and Egger's tests for 2-year overall survival was (*p* = 0.174, *p* = 0.041, respectively). Due to the small sample diameter and short follow-up time of the studies, publication bias could not be confirmed. There was no evidence of publication bias for 1-year OS (*p* = 0.734, Begg's test and *p* = 0.453, Egger's test). The results of subgroup analysis on overall survival are presented in Table 4.

## Discussion

Hepatocellular carcinoma is now the sixth most common type of cancer, and the fourth most common cause of cancer-related deaths worldwide (27), while early hepatocellular carcinoma and OS with RFA are comparable to surgical resection (28). Moreover, FI, which can apply information obtained from different imaging methods to generate excellent efficacy and safety by combining the advantages of real-time ultrasound and high resolution CT/MRI, may be more useful than ultrasound in RFA (29). Therefore, the metaanalysis systematically analyzed six studies (10, 21–23, 25, 26), comprising 1,168 patients, and found that in the RFA of hepatocellular carcinoma, FI decreased 1-year OS, and thereis no significant changes in the efficiency of ablation technology, 1–3 year LTP, 2-year OS, and complications compared to ultrasound. Notably, there was clinical heterogeneity which might affect the result due to the difference in the type of control group included in the study. Therefore, the article further compared the differences about efficacy and safety of CEUS, FI (10, 22, 26), and the differences about efficacy and safety of ultrasound and FI in RFA (21, 23, 25). When studying the efficacy and safety of FI and CEUS, there was also no significant change in the results (technical efficiency, *P* = 0.84; 1-year LTP, *P* = 0.55; 2-year LTP, *P* = 0.21; 3-year LTP, *P* = 0.63; Complications, *P* = 0.3; 1-year OS, *P* = 0.04; 2-years OS, *P* = 0.18). Since there were few studies comparing FI and ultrasound, only analyzing the complications found no significant difference in the study results (*P* = 0.52).

The high echo of the gas generated by heating immediately after ablation will greatly blur the ultrasound image of the lesion and make the next puncture difficult, while there is no the interference of vaporization in FI. In addition, when the lesion is not obvious in ultrasound examination, FI can clearly show the lesion, which helps to reduce the difficulty of surgery (30, 31). Moreover, FI has been shown to increase visibility of liver lesions, which significantly increases the confidence of operators when performing RFA (32, 33), thereby improving surgical outcomes and reducing the associated risks and complications (34). Results of our subgroup analyses corroborated these findings, as evidenced by fewer complications in studies that used FI protocol for tumors with a mean diameter <15 mm. Notably, conventional intraoperative residual tumor detection in CEUS has mainly depended on characteristic enhancement of tumors (35), while FI can show the spatial relationship between the original tumor and the ablation area (36, 37), thereby improving accuracy of evaluating intraoperative ablation edge and reducing the residual tumor. Therefore, FI may improve efficacy of ablation surgery, and expand its indications (20, 21), which is consistent with the findings of our subgroup analyses. Specifically, this technique resulted in significantly lower 1- and 2-year LTP, relative to ultrasound in FI for tumors with a mean diameter >15 mm, and the subgroup analyses also confirmed that tumor diameter was the source of heterogeneity.

However, FI has its limitations. Firstly, inherent image distortion between US and CT/MR images is inevitable, especially when patients undergo changes in position, artificial ascites, pleural effusion, or other adjuvant surgery (38). Secondly, location of subcapsular tumors represents an important factor affecting misdiagnosis after fusion image-guided HCC ablation (10). Large anatomical markers, such as the portal vein branch, cannot be used for the localization of such tumors, while the rib shadow can obscure the line of sight of the tumor. These phenomenon increase the difficulty of ablation and may affect the accuracy of FI registration. It also suggests that FI may have limited effect on improving the efficacy and safety of thermal ablation of in HCC patients. So, FI was not significantly different from ultrasound in the efficiency of ablation technology, 1–3 year LTP, and 2-year OS. But the study (39) has found that the distance between the tumor and the surrounding anatomical markers (<3 cm) can significantly reduce the surgical efficacy using FI, which has nothing to do with the location and diameter of the tumor or the patient's voluntary breathing, etc. Therefore, further research is needed to explore the limitations of FI.

The advantage of this study is that the difference in efficacy and safety between contrastive FI and ultrasound in RFA is controversial and there has not been a relevant metaanalysis. Secondly, this study evaluated the effectiveness of the ablation technique, 1–3 years of LTP, complications, and 1–2 years of OS, and performed subgroup analysis based on tumor diameter and type of control group. In addition, any bias in the review process can be minimized by conducting a comprehensive search of the literature and by following PRISMA guidelines for conducting and reporting reviews.

The study also had some limitations. Firstly, it was difficult to conduct randomization due to the nature of the intervention, so most of the included studies are cohort studies, with a few high-quality studies. Secondly, differences in geographical regions, ages, and sexes of the patients might have introduced some bias (40). Thirdly, further studies are needed to investigate the indications of FI in RFA due to inconsistent definitions for evaluating liver function and difficult lesions. Fourthly, since there are many FI schemes and FI has certain efficacy for tumors of a certain diameter (3–5 cm) (41), it is necessary to find perfect relevant studies on the differences between FI schemes and their application value.

## Conclusion

Currently, FI may play a role in improving the efficacy and safety of thermal ablation of HCC, compared to ultrasound, and may be more suitable for cases involving large lesions and difficult ablation. What is important is this study may provide a kind of research idea for the application value of FI. However, rigorous randomized controlled trials, with a larger sample diameter, are needed to validate these conclusions.

## Data Availability Statement

The original contributions presented in the study are included in the article/supplementary material, further inquiries can be directed to the corresponding author/s.

## Author Contributions

TJ, SZ, and LM contributed to the research concept and design. TJ, TG, and FG participated in literature retrieval, data collection, and data analysis. TJ, FG, SZ, and LM contributed to the drafting and review of the final manuscript. All authors read and approved the final manuscript.

## Funding

This work was supported by the National Natural Science Foundation of China (Grant No. 81601513), Young and Middle-aged High-end Talents Program of Medical Research Program of Chongqing Health and Family Planning Commission (No. 2019GDRC002), and Chongqing Science and Technology Bureau (cstc2019jcyj-msxmX0837).

## Conflict of Interest

The authors declare that the research was conducted in the absence of any commercial or financial relationships that could be construed as a potential conflict of interest.

## Publisher's Note

All claims expressed in this article are solely those of the authors and do not necessarily represent those of their affiliated organizations, or those of the publisher, the editors and the reviewers. Any product that may be evaluated in this article, or claim that may be made by its manufacturer, is not guaranteed or endorsed by the publisher.
